# Western North Pacific influences on the interannual to decadal variability of Barents-Kara Sea ice during spring

**DOI:** 10.1126/sciadv.ady7939

**Published:** 2025-12-10

**Authors:** Shutao Cao, Anmin Duan, Aiguo Dai, Chao Zhang, Ping Zhang, Yuzhuo Peng, Qi Mao

**Affiliations:** ^1^ Center for Marine Meteorology and Climate Change, State Key Laboratory of Marine Environmental Science, College of Ocean and Earth Sciences, Xiamen University, Xiamen, China.; ^2^ Department of Atmospheric and Environmental Sciences, University at Albany, State University of New York, Albany, NY 12222, USA.; ^3^ Marine Biogeochemistry Division, GEOMAR Helmholtz Centre for Ocean Research, Kiel, Germany.; ^4^ State Key Laboratory of Numerical Modeling for Atmospheric Sciences and Geophysical Fluid Dynamics (LASG), Institute of Atmospheric Physics, Chinese Academy of Sciences, Beijing, China.

## Abstract

Spring sea ice cover in the Barents-Kara Seas (BKS) exhibits notable interannual to decadal variability with spatial patterns distinct from winter. While previous studies suggest that internal climate variability dominates, the specific drivers remain to be identified. Using observations and model simulations, we reveal that atmospheric teleconnection from the western North Pacific accounts for ~26 to 40% of the interannual to decadal variability of the BKS sea ice during February-March-April. A poleward-propagating Rossby wave train, forced by divergent winds associated with the Western Pacific (WP) pattern, links WP to BKS sea ice variability by inducing lower-atmospheric circulation anomalies over the North Atlantic that enhance poleward heat and moisture transport. Furthermore, decadal shifts in the WP pattern, potentially originating from the Pacific Decadal Oscillation, have modulated the sea ice trend over the BKS, driving accelerated retreat in the 2000s and a slowdown after 2015. A projected transition of WP to its negative phase may accelerate BKS sea ice loss in the coming decade.

## Teaser

Western North Pacific atmospheric patterns influence spring Barents-Kara Sea ice variability and may speed up future ice loss.

## INTRODUCTION

In recent decades, the sea ice cover in the Barents-Kara Seas (BKS) has exhibited a pronounced long-term declining trend (*1*, *2*), accompanied by substantial variability on interannual (*3*–*7*) to decadal (*2*, *5*, *8*) timescales. Changes and fluctuations in BKS sea ice have substantial impacts not only on Arctic climate (*9*–*11*), ecosystems (*12*–*14*), and navigation activities (*15*–*17*) but also potentially on weather and climate patterns beyond the Arctic by modulating atmospheric circulations (*18*–*20*). Therefore, it is important to understand the causes of BKS sea ice variability.

Previous studies have made substantial progress in understanding the physical mechanisms of BKS sea ice variability, including both oceanic and atmospheric pathways. On the one hand, warm and salty water from the North Atlantic transports heat from the subpolar region toward the Arctic through the Barents Sea Opening (BSO), thereby influencing BKS sea ice across multiple timescales (*21*–*24*). On the other hand, many studies have also suggested that internally driven atmospheric processes play a major role in shaping sea ice variability. Among these, high-latitude atmospheric circulation modes, such as the North Atlantic Oscillation (NAO) and Arctic Dipole, can directly influence sea ice through local air-sea interactions (*2*, *3*, *7*, *8*). In addition, the anomalous downward longwave radiation induced by the transport of moisture and heat from the North Atlantic (*25*–*28*), which has been linked to sea surface temperature (SST) variability in tropical oceans (*4*, *29*, *30*), is considered a key physical process driving BKS sea ice changes. Nevertheless, these studies have primarily focused on wintertime BKS sea ice variability, while the underlying causes of springtime variability have received considerably less attention, despite the fact that its amplitude is comparable to that observed in winter (fig. S1). The spatial pattern of sea ice variability in BKS exhibits a marked change from winter to spring, with the region of greatest variability shifting from the northern to the southern Barents Sea (fig. S2). This raises essential questions regarding whether the key drivers of sea ice variability in spring differ from those in winter. A study based on large-ensemble model simulations (*31*) suggests that internal variability plays a dominant role in the multidecadal variability of spring sea ice in the Barents Sea. However, the specific drivers have yet to be identified, and the potential role of large-scale atmospheric circulation patterns over the mid- to low latitudes warrants further investigation.

The Western Pacific (WP) teleconnection pattern (*32*, *33*), featuring a north-south dipole of geopotential height anomalies with one center located over the Kamchatka Peninsula and another center of the opposite sign covering the western subtropical North Pacific region, is one of the dominant modes of atmospheric variability controlling regional climate in the North Pacific during the boreal winter and spring (*34*, *35*). Despite receiving considerably less attention than other Pacific counterparts such as the Pacific–North America (PNA) pattern and El Niño–Southern Oscillation (ENSO), the WP can not only exert a pronounced influence on the climate over the mid- to high latitudes of Eurasia and North America (*35*–*38*) but also play a crucial role in modulating the marginal sea ice in the Pacific sector of the Arctic (*39*, *40*). However, an outstanding question is whether the WP plays a role in the variability of BKS sea ice. In this study, we detected a robust linkage between the WP mode and BKS sea ice on interannual to decadal timescales during springtime and revealed the underlying physical mechanisms through analyses of observations, reanalysis, and numerical experiments.

## RESULTS

### Observed relationship between WP and BKS sea ice during springtime

**Fig. 1.**
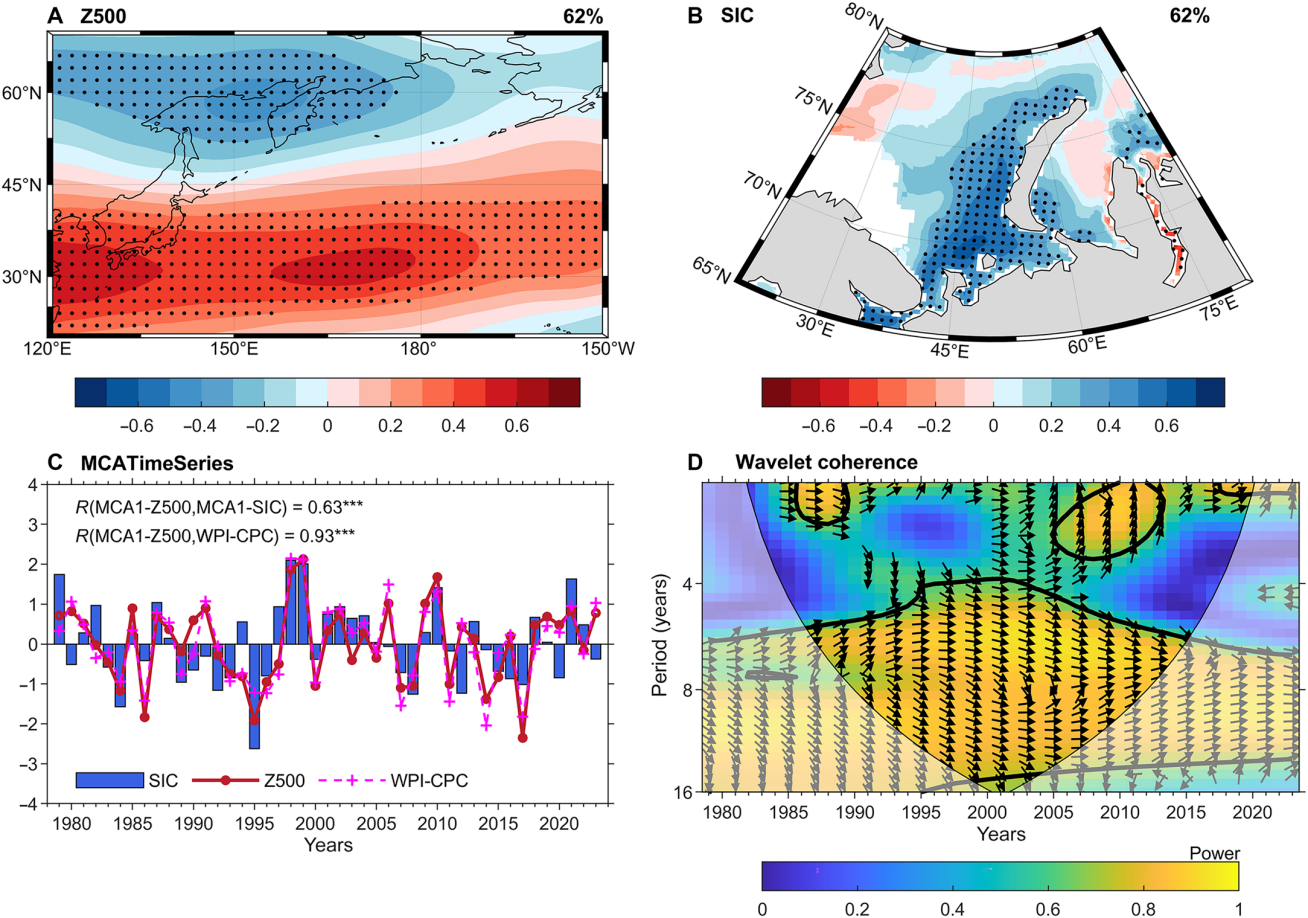
Coupled relationships between 500-hPa height (Z500) over the western North Pacific region and SIC in the BKS during FMA. (**A** and **B**) Heterogeneous correlation maps between local FMA (A) Z500 from ECMWF Reanalysis v5 (ERA5) or (B) SIC from the National Snow and Ice Data Center (NSIDC) and the temporal coefficient [shown in (C)] of the first leading MCA (MCA1) mode. (**C**) Normalized expansion coefficients for Z500 (solid line) and SIC (bar) associated with the MCA1, along with the normalized WPI (dashed line) from the Climate Prediction Center (CPC). (**D**) Wavelet analysis of the coherence between the normalized expansion coefficients of Z500 and SIC. Stippling in the shading plots indicates statistical significance at the 5% level. In (C), three asterisks next to the correlation coefficient indicate significance at the 99% confidence level. The thick black contour and arrows in (D) represent the 95% confidence level and the relative phase relationship between the time series, respectively.

Using maximum covariance analysis (MCA) (*41*), the coupled relationship between the mid-troposphere (500 hPa) geopotential height in the western North Pacific region (120°E to 150°W, 20°N to 70°N) and sea ice concentration (SIC) in BKS (20°E to 80°E, 65°N to 80°N) during early spring [February-March-April (FMA)] is revealed. The first leading MCA mode, which accounts for 62% of the squared covariance, represents a coupled pattern between the western North Pacific Z500 (Fig. 1A), which highly resembles the positive phase of the WP mode (*35*) and the positive anomaly of BKS SIC (Fig. 1B). The correlation coefficient between the MCA1 expansion coefficients for Z500 and SIC is 0.63, exceeding the 99% confidence level (Fig. 1C). In addition, a wavelet analysis of the coherence between the two time series reveals prominent variations around timescales of 4 to 12 years (Fig. 1D), indicating strong coherence on both multiannual and decadal timescales.

In addition, empirical orthogonal function (EOF) decomposition was applied to extract the spatial modes and corresponding principal components (PCs) in spring Z500 and BKS SIC (fig. S3). The results show that the second leading mode of Z500 in the northwestern Pacific region effectively characterizes the WP (fig. S3A), which is consistent with previous studies (*38*, *42*). The correlation coefficients between the expansion coefficient of MCA1-Z500 and the WP index obtained from the Climate Prediction Center (WPI-CPC; dashed line in Fig. 1C), as well as PC2-Z500 (fig. S3C), reach 0.93 and 0.84, respectively, indicating a high degree of consistency among the three WP-related indices. Therefore, we define the expansion coefficient associated with MCA1-Z500 as the WPI in this study. In a similar manner, the MCA1-SIC is designated as the SIC index (SICI) for the BKS, as the results from the MCA exhibit strong agreement with those derived from the EOF analysis (fig. S3, B and D). Considering the correlation coefficient between the MCA time series (*r* = 0.63), as well as that between SIC-PC1 and WPI-CPC (*r* = 0.51; fig. S3D), we estimate that ~26 to 40% of the interannual to decadal variability of BKS-SIC can be attributed to the WP. In addition, the MCA-derived WPI also shows a correlation of 0.51 with the BKS-averaged SIC time series (fig. S3C), underscoring the robustness and physical relevance of the WP-SIC connection. To minimize the potential subjectivity of regional selection in the MCA results, we performed an extended MCA analysis (fig. S4) using Z500 over a broader mid-latitude domain (0°E to 360°E, 20°N to 80°N). Three atmospheric circulation modes associated with BKS sea ice anomalies are identified in the extended MCA1-Z500 field (fig. S4A), resembling the Ural high (UH), the WP pattern, and the NAO, respectively. Among them, the UH-like pattern is interpreted as a response to the reduction of BKS sea ice (*43*–*45*), while the NAO-like pattern, as will be demonstrated in the next section, is shown to be a downstream response triggered by the wave train propagation originating from the WP. The correlation coefficient between the extended MCA1-Z500 time series and the WPI-CPC index (*r* = 0.77; fig. S4C) suggests that the WP pattern explains about 60% of the variance in the extended MCA1-Z500, indicating that the robust linkage between the WP pattern and BKS sea ice anomalies is not sensitive to the spatial domain selected for the MCA.

The above statistical analysis provides evidence for a significant correlation between the WP and BKS SIC. When the FMA-WP is in its positive phase, BKS SIC tends to increase, particularly along the western coast of Novaya Zemlya, and vice versa. In the following sections, the physical mechanisms behind this strong relationship are examined.

### Physical mechanisms linking WP to BKS SIC

**Fig. 2.**
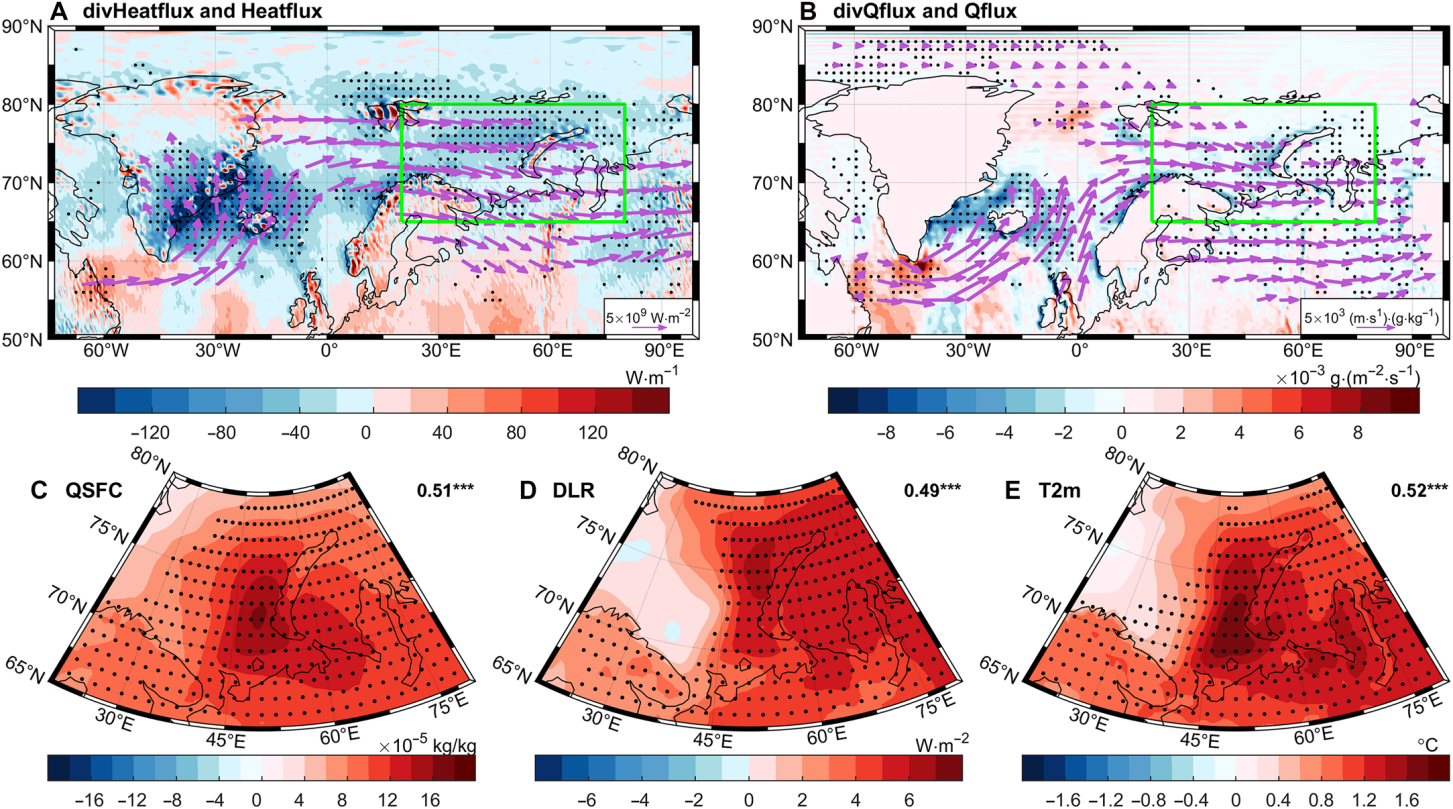
Atmospheric circulation and thermodynamic anomalies associated with the inverted (multiplied by −1) WPI during FMA. (**A**) Regression patterns of vertically integrated FMA heat flux (vectors) and its divergence (shading) onto the inverted WPI. (**B**) Same as (A) but for moisture flux and its divergence. (**C** to **E**) Regression patterns of low-tropospheric (1000 to 850 hPa) specific humidity (QSFC; C), surface DLR (D), and T2m (E) over the BKS region onto the inverted WPI. Stippling in the shading plots indicates statistical significance at the 5% level, and only significant vectors are shown in (A) to (C). The green box in (A) and (B) denotes the BKS region. The values in the top right corners of (C) to (E) represent the correlation coefficients between the corresponding thermodynamic variable averaged over the BKS region and the inverted WPI, with three asterisks indicating statistical significance at the 99% confidence level.

To reveal the physical mechanisms, we regressed atmospheric circulation and thermodynamic fields around the BKS onto the WPI (Fig. 2). All of the regression maps are multiplied by −1 to represent the anomalies associated with a negative phase of WP. Associated with the negative phase of WP, a negative anomaly of heat and moisture flux divergence is observed across the whole BKS region (shading in Fig. 2, A and B), accompanied by a positive anomaly in heat and moisture transport into the BKS (vectors in Fig. 2, A and B) from the North Atlantic, indicating anomalous convergence and accumulation of thermal energy and water vapor within the BKS. Therefore, the lower troposphere over the BKS experiences a pronounced increase in specific humidity (QSFC; Fig. 2C) and temperature [2-meter air temperature (T2m); Fig. 2E]. The increase in air temperature and water vapor, as an important greenhouse gas, will enhance the downwelling longwave radiation (DLR) over the BKS (Fig. 2D), causing sea ice to melt, which can further amplify BKS warming (Fig. 2E) by releasing heat from the ocean to the air. All of these thermodynamic processes can contribute to sea ice melting or suppression of seasonal sea ice growth. The correlation coefficients between the inverted WPI and the QSFC, DLR, and T2m over the BKS region are 0.51, 0.49, and 0.52 (all *P* < 0.01), respectively, suggesting that WP may play an important role for the interannual to decadal variations in the lower-tropospheric thermodynamic processes over the BKS region.

**Fig. 3.**
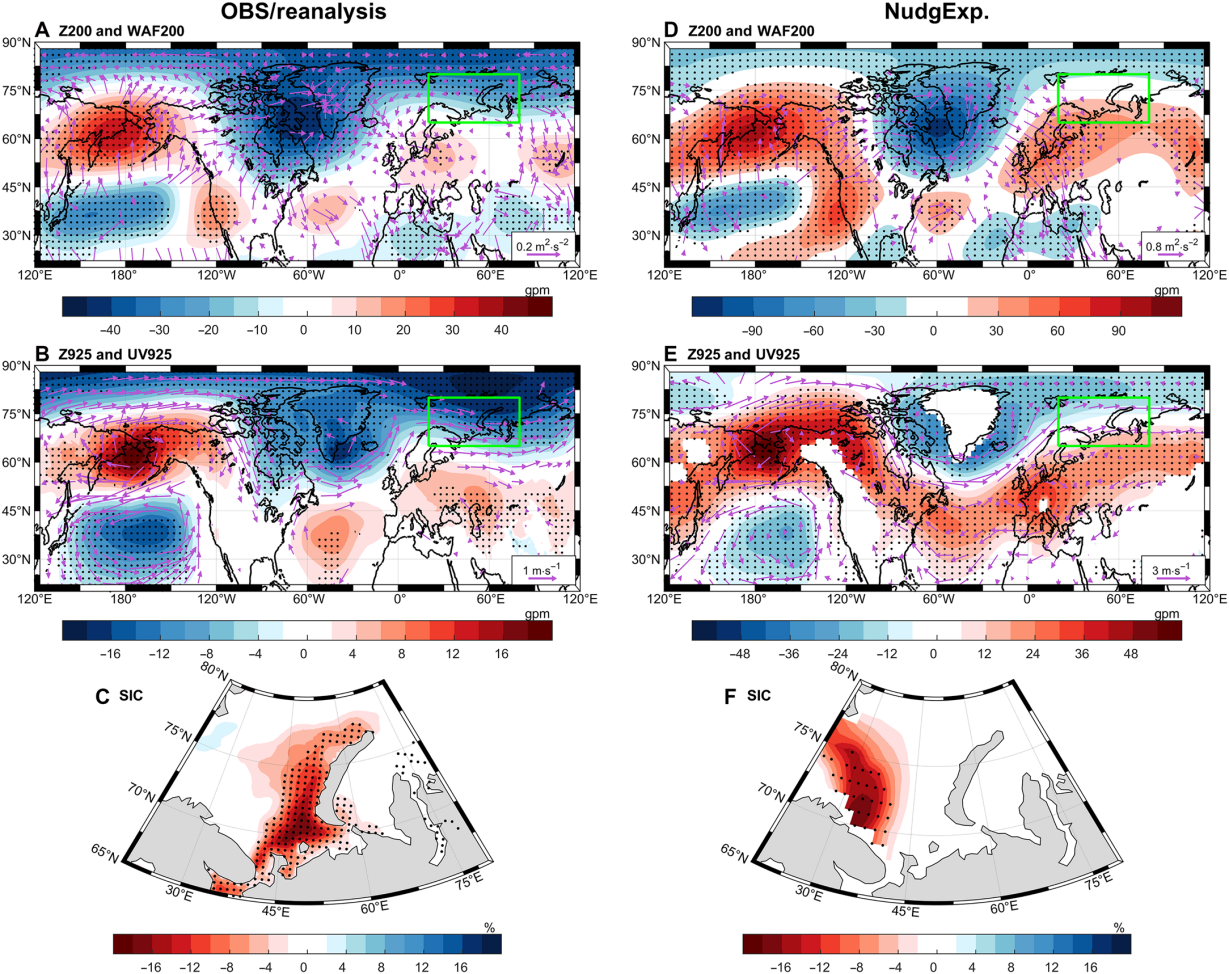
Atmospheric circulation and SIC anomalies in response to the WPI in both reanalysis (ERA5) or observations (OBS; NSIDC) and nudging simulations during FMA. (**A**) Regression patterns of FMA 200-hPa geopotential height anomalies (Z200; shading) and WAF (WAF200; vectors) from ERA5 onto the inverted WPI. (**B**) Regression patterns of FMA 925-hPa geopotential height (Z925; shading) and horizontal wind anomalies (UV925; vectors) from ERA5 onto the inverted WPI. (**C**) Regression patterns of SIC anomalies onto the inverted WPI. (**D** to **F**) Same as (A) to (C) but for the Community Earth System Model version 2 (CESM2)–simulated response to WPI in the nudging experiments (NGNEG minus NGPOS ensemble mean difference). Stippling in the shading plots indicates statistical significance at the 5% level, and only significant vectors are shown in (B) and (E). The unit of geopotential height is geopotential meters (gpm). The green boxes denote the BKS region.

An obvious question is how this teleconnection between WP and BKS SIC is generated, likely through an atmospheric bridge. The responses of atmospheric circulations and sea ice to the WP pattern based on observations [National Snow and Ice Data Center (NSIDC)], reanalysis [ECMWF Reanalysis v5 (ERA5)], and nudging simulations are shown in Fig. 3. In the ERA5 reanalysis, a distinct Rossby wave train, consisting of four geopotential height anomaly centers propagating poleward, is observed in the upper troposphere (Fig. 3A). Wave activity flux (WAF; vectors in Fig. 3A) and Rossby wave source (RWS; fig. S5D) analysis reveal that the wave energy originates from the western North Pacific region, with the RWS mainly attributed to the atmospheric absolute vorticity advection term (fig. S5, E and F) induced by divergent wind anomalies (fig. S5C). This upper-level divergence anomaly is shown to result from the anomalous convergence (fig. S5A) and associated upward motions (fig. S5B) in the lower troposphere. The meridional vertical circulation anomalies (fig. S6) also reveal ascending motion from the lower to the upper troposphere within the 20°E-to-40°E region, accompanied by divergence at the upper level.

The WP-related lower-tropospheric geopotential height anomalies (shading in Fig. 3B) exhibit a pattern similar to that of 200-hPa height field, suggesting a barotropic structure, with a resemblance to that of the NAO in the Atlantic-Arctic region. The correlation coefficients between the NAO index and the WPI and BKS SICI are 0.40 and 0.35 (*P* < 0.05; fig. S7), respectively, which, although lower than the correlation between the WPI and SICI, suggests that the NAO may act as a bridging mechanism. The corresponding lower-tropospheric horizontal wind anomalies (vectors in Fig. 3B) facilitate the transport of heat and moisture (Fig. 2, A and B) from the North Atlantic to the Arctic (*4*, *26*, *46*), particularly toward the BKS, which leads to sea ice melting (Fig. 3C) in the BKS through increased DLR and local thermodynamic processes (Fig. 2, C to E).

### Response of atmospheric circulation and BKS Sea ice to WP in numerical simulations

In the above, we proposed a plausible mechanism explaining how WP can influence BKS sea ice via the atmospheric bridge based on regression analysis of reanalysis and observational data. However, it is challenging and not entirely convincing to determine causality solely based on data diagnosis because the atmosphere and sea ice are coupled with each other via sea ice–air two-way interactions (*47*) and complex feedback mechanisms (*48*, *49*). In other words, it remains uncertain whether the increase in surface specific humidity and temperature over BKS (Fig. 2, C and E) is driven by the teleconnection effect of WP or just a result of local sea ice reduction. To address this question, we conducted two sets of numerical experiments (see Materials and Methods) using the Community Earth System Model version 2 (CESM2), in which the atmospheric component [Community Atmosphere Model version 6 (CAM6)] was nudged to the wind fields corresponding to the negative (NGNEG) and positive (NGPOS) phases of the WP pattern, respectively. The WP-induced changes in the climate system can be isolated by differencing ensemble means of the two sets of experiments (NGNEG minus NGPOS), with the results shown in Fig. 3 (D to F).

It can be seen that the nudging simulations demonstrate a response to the WP highly consistent with observations. These responses include a barotropic Rossby wave train originating from the western North Pacific with four centers (Fig. 3, D and E), anomalous southwesterly winds in the lower troposphere from the North Atlantic to the Arctic (Fig. 3E), and the resulting negative SIC anomalies in the BKS region (Fig. 3F), although the exact location of the SIC anomalies is shifted to the west due to the mean bias of the ice edges in the CESM2 (fig. S8). In the additional sensitivity experiments where the nudging coefficients were reduced to smaller values, the patterns of large-scale atmospheric circulation and sea ice anomalies remain consistent with those in the original experiments (fig. S9), except for a reduced amplitude of the sea ice anomalies in the weak-nudging experiments (fig. S9G), indicating that the simulated responses to the WP pattern are robust features and are insensitive to the choice of nudging strength. The consistency between the simulations and observations or reanalysis provides convincing evidence for the atmospheric teleconnection through which the spring WP can have a remote impact on BKS sea ice.

Several studies (*22*, *50*, *51*) have suggested that atmospheric circulation anomalies in the North Atlantic region can influence Barents Sea ice cover through oceanic pathways by modulating ocean heat transport (OHT) across the BSO. This raises the question of whether the NAO-like response over the North Atlantic, induced by the WP pattern, contributes to sea ice anomalies in the BKS through oceanic pathways. Responses of the horizontal ocean heat flux (fig. S10A) indicate that atmospheric circulation anomalies over the North Atlantic associated with the negative phase of the WP drive anomalous eastward ocean currents, facilitating the accumulation of warm water primarily along the western coast of the Scandinavian Peninsula, with part of the flow entering the Barents Sea through the BSO (text S1 and fig. S10B). Notably, while observations, reanalysis, and other model simulations typically report OHT values of 50 to 70 TW (*22*, *50*, *52*), our simulation yields only 10 to 20 TW, which is consistent with the extensive sea ice cover in our simulation (fig. S8). However, the sea ice budget analysis for concentration, thickness (fig. S10, C and D), and volume (text S2 and fig. S10E) shows that basal melting due to ocean-ice heat flux contributes to only 14% of the total sea ice volume tendency anomalies, indicating that the oceanic pathway is not the primary driver of sea ice changes over the timescales considered in this study. This finding is anticipated, as previous studies have shown that, due to the ocean’s thermal inertia, the sea ice response to OHT typically lags by several months to 2 years (*2*, *21*, *50*, *53*). In addition, we note that the simulated sea ice anomalies are primarily driven by ice drift (fig. S10, D and E) associated with the surface wind anomalies (Fig. 3E), while thermodynamical processes partially compensate for the sea ice loss induced by dynamical effects through the ice thickness-growth rate feedback (*54*), resulting in opposite patterns of the SIC budget (fig. S10, C and D). This contrasts with the mechanism revealed by observations and reanalysis, where sea ice anomalies are mainly attributed to enhanced downward longwave radiation induced by increased moisture transport. This discrepancy can largely be attributed to biases in the model’s simulation of the sea ice climatology, specifically a westward shift of the ice edge (fig. S8, A and B), which creates favorable conditions for wind-driven sea ice drift. This implies that the manner in which atmospheric circulations affect sea ice is strongly influenced by the sea ice’s baseline state, underscoring the vital importance of improving sea ice representation in climate models under the background of ongoing climate changes.

## DISCUSSION

**Fig. 4.**
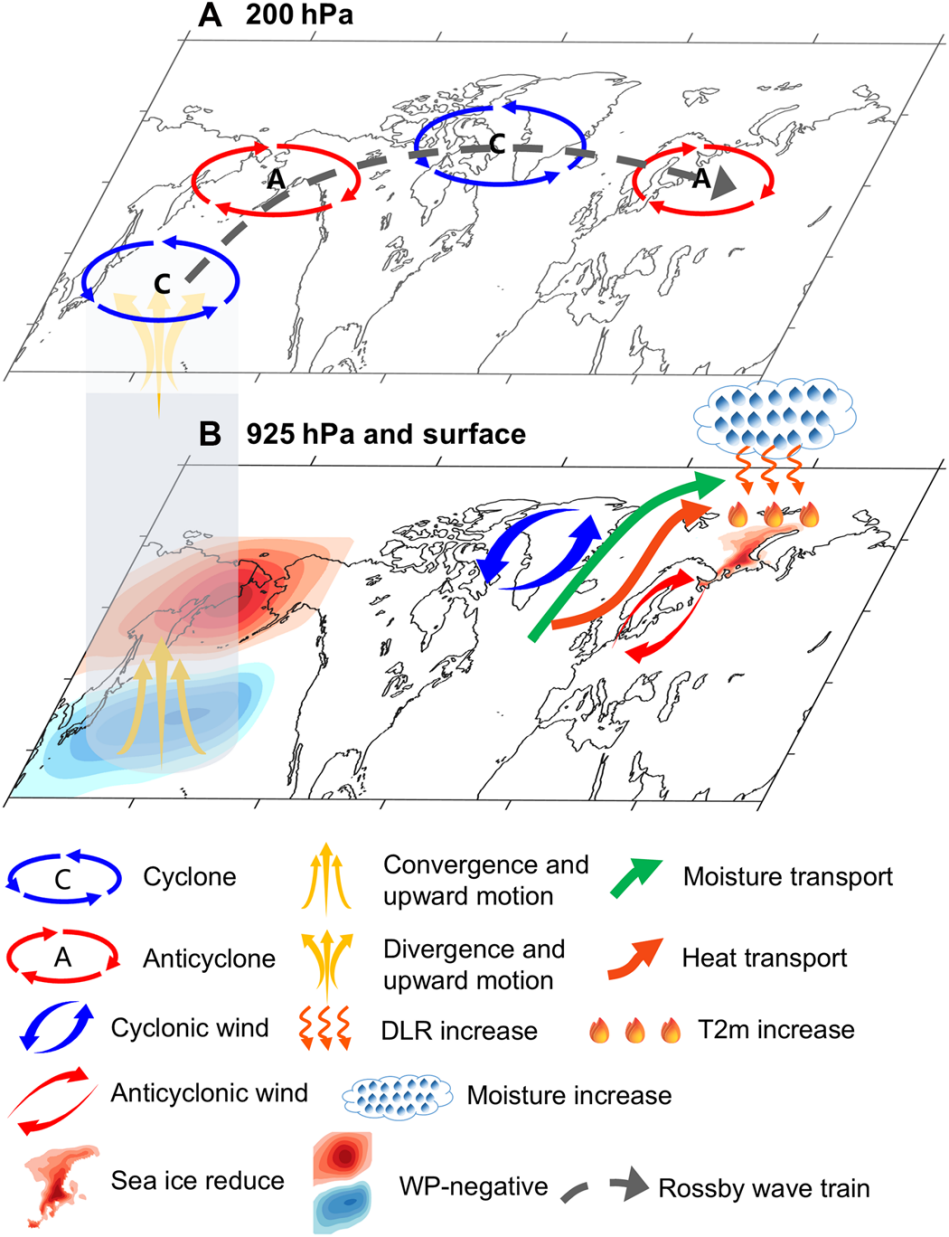
Schematic diagram illustrating the physical processes by which the WP pattern influences sea ice over the BKS in spring in the upper troposphere and near the surface. (**A**) The upward motion and associated divergent wind anomalies in the upper troposphere excite a Rossby wave train characterized by four geopotential height anomaly centers. (**B**) The anomalous atmospheric circulation in the lower troposphere exhibits a barotropic structure consistent with that in the upper troposphere, characterized by a positive NAO-like pattern over the North Atlantic, which facilitates the transport of moisture and heat toward the BKS region, increases downward longwave radiation and surface temperature, and ultimately contributes to sea ice reduction.

Sea ice variation in the BKS can have substantial impacts on regional and global climate change, Arctic routes, and the related economic benefits. Substantial progress has been made in previous studies in understanding the drivers of BKS sea ice variability during winter, and a teleconnection mechanism linking it to ENSO has been established (*4*, *6*). In spring, however, when ENSO enters its decaying phase, the spatial pattern of sea ice variability exhibits marked changes, and its linkage to mid- and low-latitude climate factors remains largely unexplored. In this study, we further reveal that an atmospheric mode over the western North Pacific, i.e., the WP, acts as an important driver of the interannual to decadal variability of BKS sea ice in springtime. The teleconnection mechanism bridging WP and BKS sea-ice variations is depicted in Fig. 4. During the negative phase of WP, a barotropic-coupled atmospheric column develops over its southern anomalous center, characterized by the convergence (divergence) in the lower (upper) troposphere with vertically coherent ascending motion. The upper-level divergence anomalies, acting as an RWS, induce anomalous vorticity forcing and subsequently trigger a stationary Rossby train that propagates toward the Arctic. The wave train consists of four geopotential height anomaly centers, exhibiting an equivalent barotropic structure, with the anomalous low-level winds resembling the positive phase of the NAO over the North Atlantic, which facilitates the transport of warm moist air from the North Atlantic into the BKS region and subsequently leads to sea ice melting through increased downward longwave radiation and local positive feedbacks. While previous studies have emphasized the role of the NAO in connecting mid- to low-latitude variability with Arctic climate, our study reveals a distinct mechanism. Notably, a study by Ding *et al.* (*29*) identifies a Rossby wave train originating from the tropical Pacific that contributes to warming over northeastern Canada and Greenland, in which the NAO also plays a crucial role. Although the NAO serves as a key mediator in both studies, the mechanisms involved differ between their study and ours. Adiabatic subsidence associated with the negative phase of the NAO is identified as the primary driver of Arctic warming in their work, whereas our study highlights moisture and heat transport linked to the positive phase of the NAO as the key physical process. Furthermore, our study emphasizes the impact of Rossby wave trains originating from mid-latitude atmospheric variability, in contrast to forcings from tropical oceans (*29*, *30*), on Arctic sea ice, introducing additional insights into the ongoing discussion on mid-latitude–Arctic interactions within the scientific community.

**Fig. 5.**
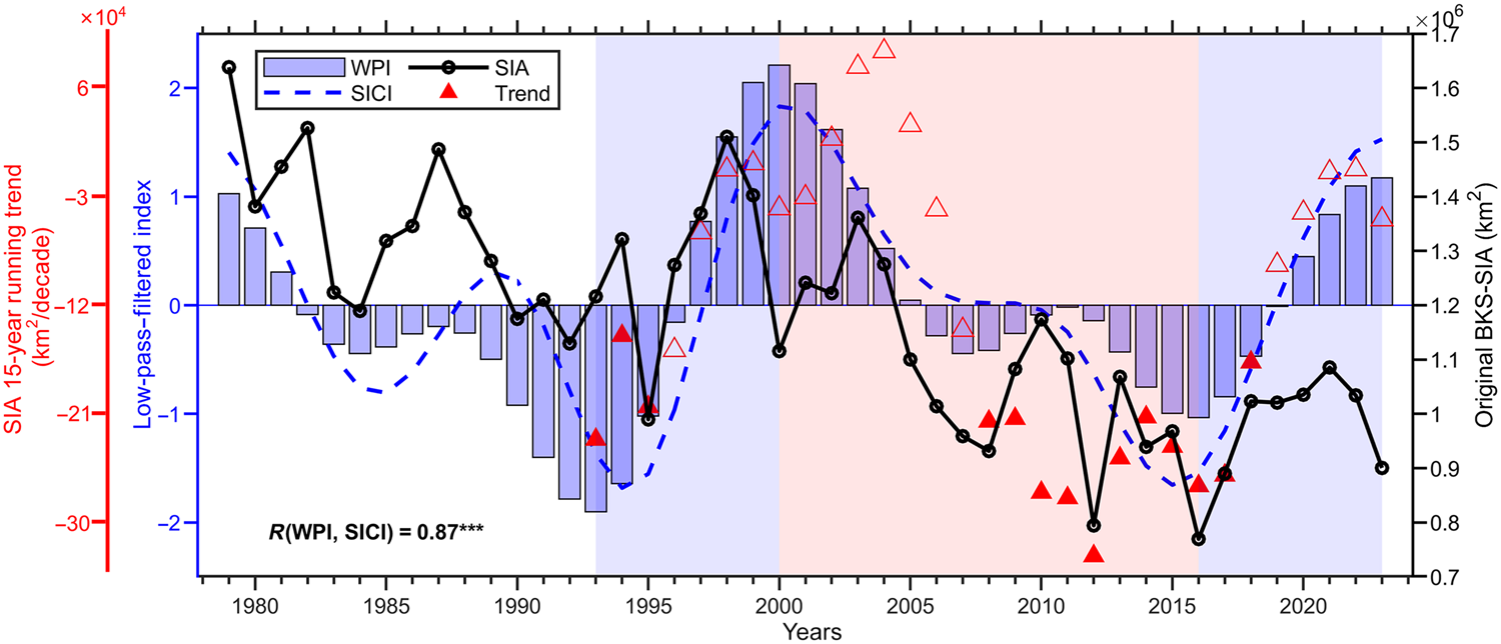
The connections between WPI and BKS SIC during FMA on decadal timescales. The blue bar and dashed line represent the WPI and BKS SICI after a 9-year low-pass filtering, respectively, while the black solid line indicates the raw sea ice area (SIA) of the BKS. The red triangles depict the sliding trend of the BKS SIA using the preceding 15-year period as the window, with filled triangles denoting trends that passed the 95% significance test. The blue-shaded areas indicate periods when the WPI transitioned from a negative to a positive phase, specifically from 1993 to 2000 and from 2016 to 2023. Conversely, the red-shaded area marks the transition from a positive to a negative WPI phase, occurring between 2000 and 2016. The correlation coefficient between decadal variations of WPI and SICI is shown in the lower-left corner, with three asterisks indicating statistical significance at the 99% confidence level.

Arctic sea ice changes over the past few decades result from the combined effects of internal variability and external forcings. Figure 5 shows the relationship between BKS-SIC and WP on decadal timescales. The 15-year sliding trend of BKS sea ice area tracks consistently with the WPI fluctuations, indicating that WP can either amplify or offset the externally forced sea ice loss on the decadal timescale, thereby accelerating (2000 to 2015) or decelerating (1994 to 2000 and 2015 to 2023) decadal sea ice decline. An upcoming shift of the WP to its negative phase could further enhance BKS sea ice decline in the next decade. The correlation coefficient between the decadal variations of WPI and SICI is 0.87, which is consistent with the previous finding that spring internal variability accounts for ~75% of the decadal variability in Barents SIC (*31*). Notably, the recent slowdown of sea ice loss in both ice thickness and concentration over the BKS has also been documented in observations (*55*). This strong decadal-scale coherence between the WP and BKS sea ice highlights the potential role of atmospheric circulation modes in modulating externally forced trends. To better interpret this relationship and assess its broader climate implications, it is crucial to clarify the physical origin of the WP pattern itself, especially on the decadal timescale. In general, the WP pattern has been attributed to both internal dynamical processes and forcings from oceanic or terrestrial surface variability. From a dynamical perspective, one view emphasizes the WP as an intrinsic mode of internal atmospheric variability, where the interaction between synoptic-scale eddies and the background mean flow plays a crucial role in the development and maintenance of the WP pattern (*56*, *57*). An energetic analysis of the monthly WP pattern indicates that baroclinic energy conversion from the climatological-mean flow constitutes the most effective mechanism for its maintenance (*58*). A recent study also highlights the role of baroclinic energy conversion in offsetting the available potential energy loss of the springtime WP pattern induced by transient eddies and diabatic heating (*35*). On the other hand, several studies have suggested that the WP pattern can be triggered or modulated by large-scale ocean-atmosphere modes over the Pacific or Atlantic (*34*, *59*–*62*), which is essential for interpreting its decadal variations, as the atmosphere alone is generally incapable of sustaining variability on such long timescales due to the relatively short memory. Previous studies indicate that the Pacific Decadal Oscillation (PDO) plays a crucial role in the formation and maintenance of decadal-to-interdecadal atmospheric circulation anomalies over the North Pacific (*59*, *63*, *64*). Regression of SST anomalies onto the WPI reveals a horseshoe-shaped pattern over the North Pacific (fig. S11A) that closely resembles the negative phase of PDO (fig. S11B). The correlation coefficient between the decadal components of the inverted PDO index and WPI reaches 0.75 (fig. S11C), suggesting that the PDO may serve as a potential driver of the decadal variations of the WP pattern. In the future, the physical link among the PDO, WP, and BKS-SIC deserves further study.

Among the spring months, the selection of FMA was motivated by the fact that the correlation between the WP pattern and BKS-SIC is strongest during this period (fig. S12). Notably, significant correlations between WP and BKS-SIC are also observed in other spring months (fig. S12, D to I). In contrast, no statistically significant relationship between WP and BKS-SIC is observed during summer, autumn, or winter. This seasonal dependence is physically reasonable and does not undermine the robustness of the mechanism. During summer and autumn, the amplitude of the WP pattern is typically weak, resulting in limited teleconnection climate effects. In winter, the WP pattern is strongly modulated by other large-scale climate patterns such as ENSO and the PNA pattern (*34*, *60*), which reach their peak intensity during this season and exert widespread climatic effects, potentially obscuring the expression of WP-related signals over the BKS region. Therefore, the springtime WP-SIC linkage identified in this study represents a physically plausible, seasonally constrained, and statistically robust teleconnection. Rather than limiting the importance of the mechanism, its confinement to spring highlights the importance of seasonal context in understanding cross-latitude climate interactions and the timing of their downstream impacts on the Arctic climate system.

## MATERIALS AND METHODS

### Observational and reanalysis data

Monthly SIC data used in this study were obtained from the climate data record of passive microwave sea ice concentration version 4 (*65*) from the National Snow and Ice Data Center (NSIDC), covering the period from 1979 to 2023. Monthly SST from the Hadley Centre Sea Ice and Sea Surface Temperature (HadISST) dataset (*66*) was used to calculate the PDO index. The monthly atmospheric data used in this study were taken from ERA5 (*67*) of the European Centre for Medium-Range Weather Forecasts, including geopotential height, zonal and meridional winds, vertical velocity, specific humidity, air temperature, and downward longwave radiation flux. All data were linearly detrended to focus on interannual to decadal variations. In addition, the 6-hourly zonal and meridional winds on model levels from ERA5 were used as target data to constrain the fields in the nudging experiments.

### Statistical analysis

The MCA, performed by singular value decomposition of the covariance matrix between two fields (*41*), was used to identify the coupled modes of variability between 500-hPa geopotential height (Z500) of the western North Pacific region (120°E to 150°W, 20°N to 70°N) and SIC in the BKS (20°E to 80°E, 65°N to 80°N) during early spring (FMA), with the former commonly used to characterize the WP pattern. The expansion series obtained by projecting the leading pair of singular vectors onto the original fields is used to define the WPI and SICI. In addition, we conducted a comparison of the WPI derived from the MCA with that provided by the National Oceanic and Atmospheric Administration CPC, and the results demonstrated a high degree of consistency between them.

The horizontal WAF (denoted as *W*) is used in this study to reflect the energy propagation of the quasistationary Rossby waves. Following (*68*), it can be expressed in terms of the following equation

$$W=\frac{p}{2|U|}\left[\begin{array}{c} U\left(\varphi_{x}^{\prime 2}-\varphi^{\prime }\varphi_{\mathrm{xx}}^{\prime }\right)+V\left(\varphi_{x}^{\prime }\varphi_{y}^{\prime }-\varphi^{\prime }\varphi_{\mathrm{xy}}^{\prime }\right)\\ U\left(\varphi_{x}^{\prime }\varphi_{y}^{\prime }-\varphi^{\prime }\varphi_{\mathrm{xy}}^{\prime }\right)+V\left(\varphi_{y}^{\prime 2}-\varphi^{\prime }\varphi_{\mathrm{yy}}^{\prime }\right) \end{array}\right]$$ (1)

in which *p* indicates the pressure, ***U*** = (*U*, *V*) represents the zonal and meridional components of the basic flow, and *|U|* indicates the horizontal wind speed. The subscripts and prime symbols represent the partial derivatives and the time anomaly, respectively.

The linearized barotropic RWS (denoted as *S*) is also calculated to interpret the generation of Rossby waves, which can be expressed using the following equation (*69*)

$$S=-\nabla_{H}\cdot \left[u_{\chi }^{\prime }\left(f+\bar{\zeta }\right)\right]-\nabla_{H}\cdot \left[{\bar{u}}_{\chi }\zeta^{\prime }\right]$$ (2)

where ***u*** = (*u, v*) is the zonal and meridional winds and *f* and ζ are the planetary vorticity and relative vorticity, respectively. The symbol ∇_H_ represents the horizontal gradient. χ represents the divergent component of the wind field. The overbar and prime symbols represent the climatology and anomalies, respectively.

Other commonly used statistical methods are also used, including correlation, regression, composite, and EOF analyses. A student’s *t* test (*70*) is used to examine the statistical significance of the results for regression, correlation, and the difference of composite means. The significance level is set at 5% unless otherwise stated. The effective degrees of freedom (*71*–*73*) for Student’s *t* tests can be calculated as follows

$$\frac{1}{N^{\text{eff}}}\approx \frac{1}{N}+\frac{2}{N}\sum_{j=1}^{N}\frac{N-j}{N}\rho_{\mathrm{XX}}\left(j\right)\rho_{\mathrm{YY}}\left(j\right)$$ (3)

where *N* denotes the sample size and ρ*_XX_*(*j*) and ρ*_YY_*(*j*) are the autocorrelations of two sampled time series *X* and *Y*, respectively, at time lag *j*.

### Model simulations

To investigate the physical mechanisms by which the WP influences BKS sea ice, we use CESM2.1.3 (*74*) to conduct two sets of nudging experiments. As a fully coupled climate model, CESM2 can provide state-of-the-art simulations of the Earth’s past, present, and future climate states. The atmosphere component is CAM6 and run on a finite volume dynamical core with horizontal resolution of ~1.9° by 2.5° and 32 vertical levels. The land component uses the same grid as the atmosphere, while the ocean and sea ice components are run on the displaced pole gx1v7 grid with ~1° spacing. Nudging experiments provide an approach to “replay” the observed climate variability by constraining some variables to observations over a given region while allowing others to evolve freely; it has been widely applied to study impacts of internal atmospheric modes on Arctic sea ice variability (*75*–*78*). We nudge the model to ERA5 horizontal winds following the methodology from (*75*)

$$\frac{\mathrm{dx}}{\mathrm{dt}}=F\left(x\right)+F_{\text{nudge}}$$

$$F_{\text{nudge}}=\frac{\alpha }{\tau }\left[O\left(t_{\text{next}}^{\prime }\right)-x\left(t\right)\right]$$ (4)

where *x*(*t*) represents one of the model’s prognostic variables at the model time step *t* and *F*(*x*) represents the model’s internal tendency without applying nudging. The nudging term, *F*_nudge_, is determined by the normalized nudging coefficient α (zero for no nudging and one for full nudging), the difference between the target analysis and the model’s current state $\left[O\left(t_{\text{next}}^{\prime }\right)-x\left(t\right)\right]$, and the relaxation timescale τ.

In this study, our nudging experiments nudge the model’s zonal and meridional winds in the western North Pacific region (120°E to 150°W, 20°N to 70°N) from the low troposphere (850 hPa) to the top of the atmosphere. At the edges of both vertical and horizontal nudging windows, we have set buffer zones to ensure a smooth transition from the nudging to no-nudging domains. The nudging region selected for this study is based on robust observational evidence linking circulation anomalies in this sector to downstream effects on sea ice in the BKS. We recognize that nudging imposes a constraint on large-scale circulation patterns, particularly along the jet stream, potentially suppressing the natural evolution of circulation in remote regions during spring. Although the model setup inevitably simplifies the complexity of real-world mid-latitude interactions where components coevolve more freely, nudging remains a widely used and effective method for identifying causality in the climate system (*75*–*78*), and future efforts to assess its limitations and enhance its representation of complex physical processes are important. The timestep of CAM6 is set to 30 min, and nudging is applied at each timestep by linearly interpolated nearby target values to current model time. The nudging coefficient, which determines the intensity of nudging, is set to 0.8, implying a relatively large nudging amplitude. The details of nudging experiments design can be seen in fig. S13 and table S1. We first run a 100-year control experiment (CLIM-CTRL) to get the model’s climatology. The nudging target values of zonal and meridional winds are then constructed as the sum of model climatology and ERA5 reanalysis anomalies. In contrast to directly using reanalysis as nudging target data, this approach avoids modifying the model climatology (*79*). Thirty members of time-slice experiments were run for NGPOS and NGNEG, respectively, branching from 1 February of each year of the past 30 years of CLIM-CTRL and integrated for 3 months for each member. The difference between the NGNEG and NGPOS ensemble mean can be considered as the model’s response to the wind pattern for the different phases of WP. To demonstrate the robustness of the BKS sea ice response to the WP pattern with respect to the choice of nudging strength, two additional pairs of sensitivity experiments were conducted using a moderate (0.6) and a weak (0.4) nudging coefficient, namely MDNG and WKNG, respectively, while keeping all other configurations identical to those in NGNEG and NGPOS. Because of limitations of computational resources, these additional experiments were run with only 10 ensemble members each, branching from 1 February of each year in the past 10 years of CLIM-CTRL. For all the experiments, we set external forcing as constant values at the level of year 2000, which is close to the climatology mean of 1979 to 2023.

The control experiment successfully captures the strong variability of spring SIC in the Barents Sea region (fig. S8D), albeit with a westward shift in location (fig. S8, C and D). This discrepancy arises because, under the greenhouse forcing set at the year 2000 level, the simulated climatological mean state of sea ice differs from observations (fig. S8, A and B). Nevertheless, this does not affect the response of SIC to WP as presented in the results of nudging experiments.
