# Supplementary material for: Western North Pacific influences on the interannual to decadal variability of Barents-Kara Sea ice during spring
Source: Sci Adv. 2025 Dec 10;11(50):eady7939. doi: 10.1126/sciadv.ady7939 (PMC12694036; doi:10.1126/sciadv.ady7939)
Supplement: Supplementary file 1 — Supplementary text S1 and S2 Figs. S1 to S13 Table S1 [file sciadv.ady7939_sm.pdf]

Science Advances
AAAS

Supplementary Materials for

Western North Pacific influences on the interannual to decadal variability of Barents-Kara Sea ice during spring

Shutao Cao *et al.*

Corresponding author: Anmin Duan, amduan@xmu.edu.cn

Sci. Adv. **11**, eady7939 (2025)
DOI: 10.1126/sciadv.ady7939

This PDF file includes:

Supplementary text S1 and S2
Figs. S1 to S13
Table S1

Text S1: Calculation of ocean heat transport (OHT) across the Barents Sea Opening (BSO)

Ocean heat transport (OHT) across the Barents Sea Opening (BSO) is computed by vertically integrating the advective heat flux across a section perpendicular to the mean flow, extending from the ocean surface down to a depth of 300 meters. The section is defined along 20°E between 71°N and 77°N, approximately representing the geographic location of the BSO, a critical gateway for Atlantic water inflow into the Arctic. The OHT is computed as

$$OHT = \int_S \rho c_p U(T - T_{ref}) dS,$$

Where ρ = 1025 kg m^{-3} and c_p = 4000 J K^{-1} kg^{-1} are the constant density and heat capacity of seawater, respectively; U is the ocean velocity normal to the BSO section; T is the potential temperature, T_{ref} is the reference temperature which is set to 0 °C. The resulting OHT is expressed in Watts (*W*), quantifying the total heat flux carried by ocean currents through the BSO. The variables used to calculate OHT include the zonal ocean velocity (*UVEL*) and potential temperature (*TEMP*) which obtained from CESM2 nudging experiments.

Text S2: Calculation of sea ice volume (SIV) budget

In order to assess the relative contributions of different physical processes to the sea ice changes over the Barents-Kara Seas (BKS), we diagnose the sea ice volume budget terms using output from the sea ice component of CESM. The total changes of SIV can be attributed to changes from thermodynamics and dynamics:

$$\frac{dSIV}{dt} = thermo + dyn,$$

where the SIV tendency due to thermodynamics is the sum of contributions from basal, surface, lateral growth, and frazil ice formation:

$$thermo = basal + surface + lateral + frazil$$

The SIV tendency due to dynamics refers to the convergence caused by sea ice drift. The variables used to calculate the sea ice volume budget terms include basal melting (*meltb*), congelation ice growth (*congel*), surface melting (*meltt*), snow-ice formation (*snoice*), evaporative water flux (*evap*), lateral melting (*meltl*), and volume tendency due to dynamics (*dvidtd*). All variables are obtained from the sea ice component of CESM2 and are expressed in units of cm/day. Each variable is multiplied by the corresponding grid cell area (*tarea*) and weighted by sea ice concentration (*aice*) to obtain the actual contribution of that variable to the SIV tendency.

As shown in Fig. 3F, the most pronounced sea ice response simulated by CESM2 is located in the western Barents Sea (20–45°E, 65–80°N). To assess the main contributors to sea ice changes, we perform a spatial averaging of the sea ice volume budget components over this region, with the results shown in fig. S10E.

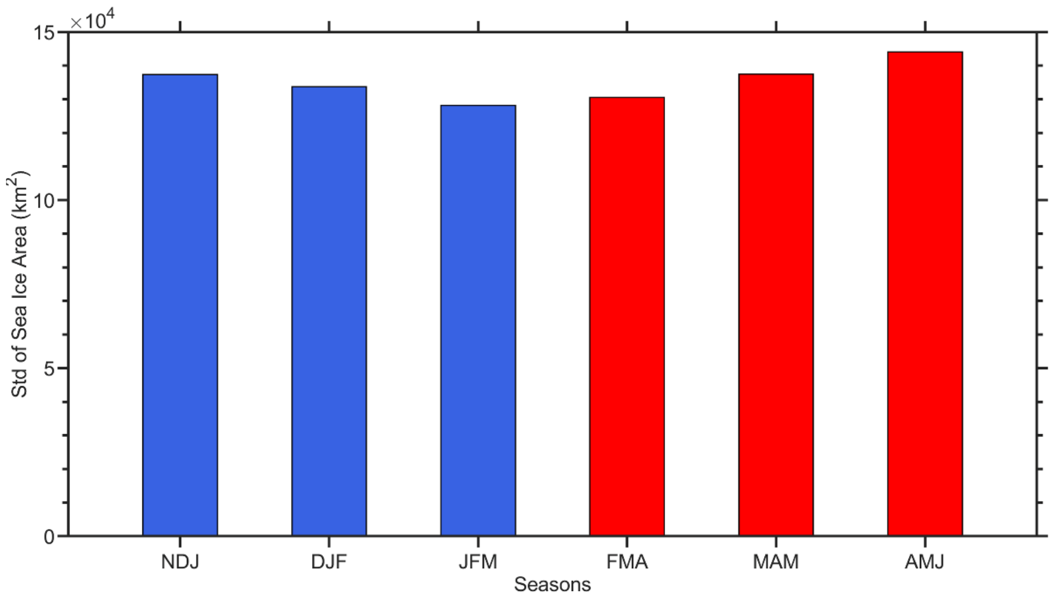

Fig. S1. Standard deviation of sea ice area in the BKS across winter (blue) and spring (red) seasons during 1979–2023.

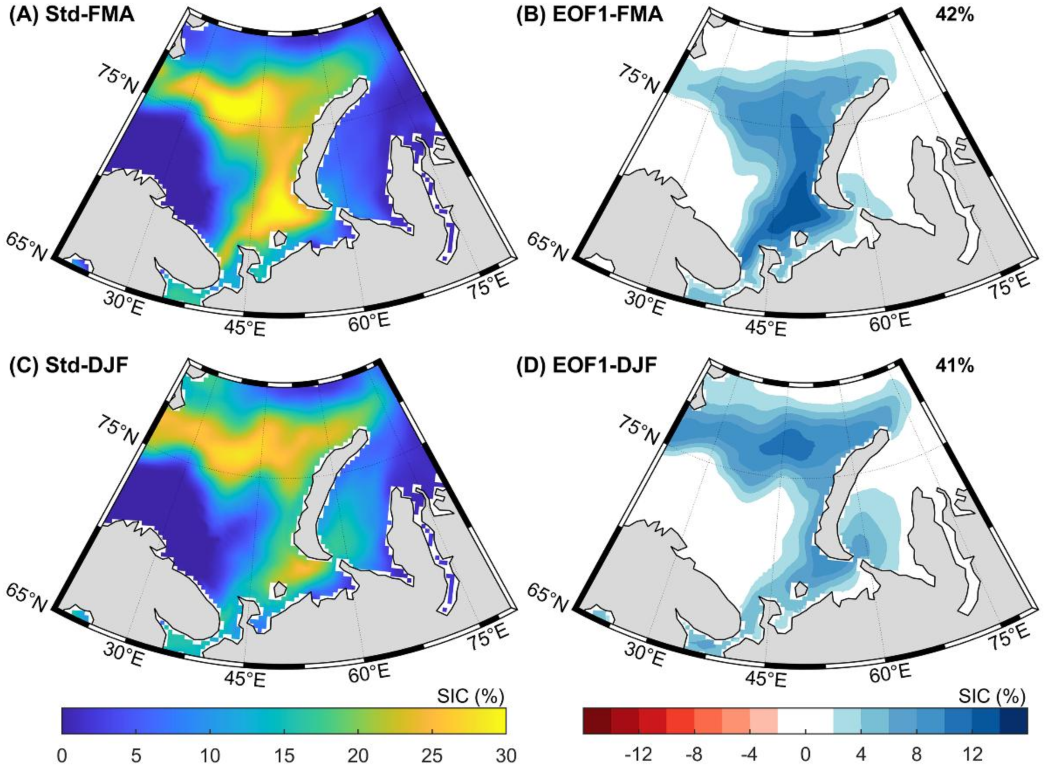

Fig. S2. Spatial patterns of sea ice concentration (SIC) variability in the BKS during spring (A, B) and winter (C, D). Panels **(A)** and **(C)** show the standard deviation of SIC, **(B)** and **(E)** represent the spatial patterns of EOF1, respectively. The explained variance of EOF1 is indicated in the upper right corner of panels (B), (D). FMA and DJF refer to February-March-April and December-January-February, respectively.

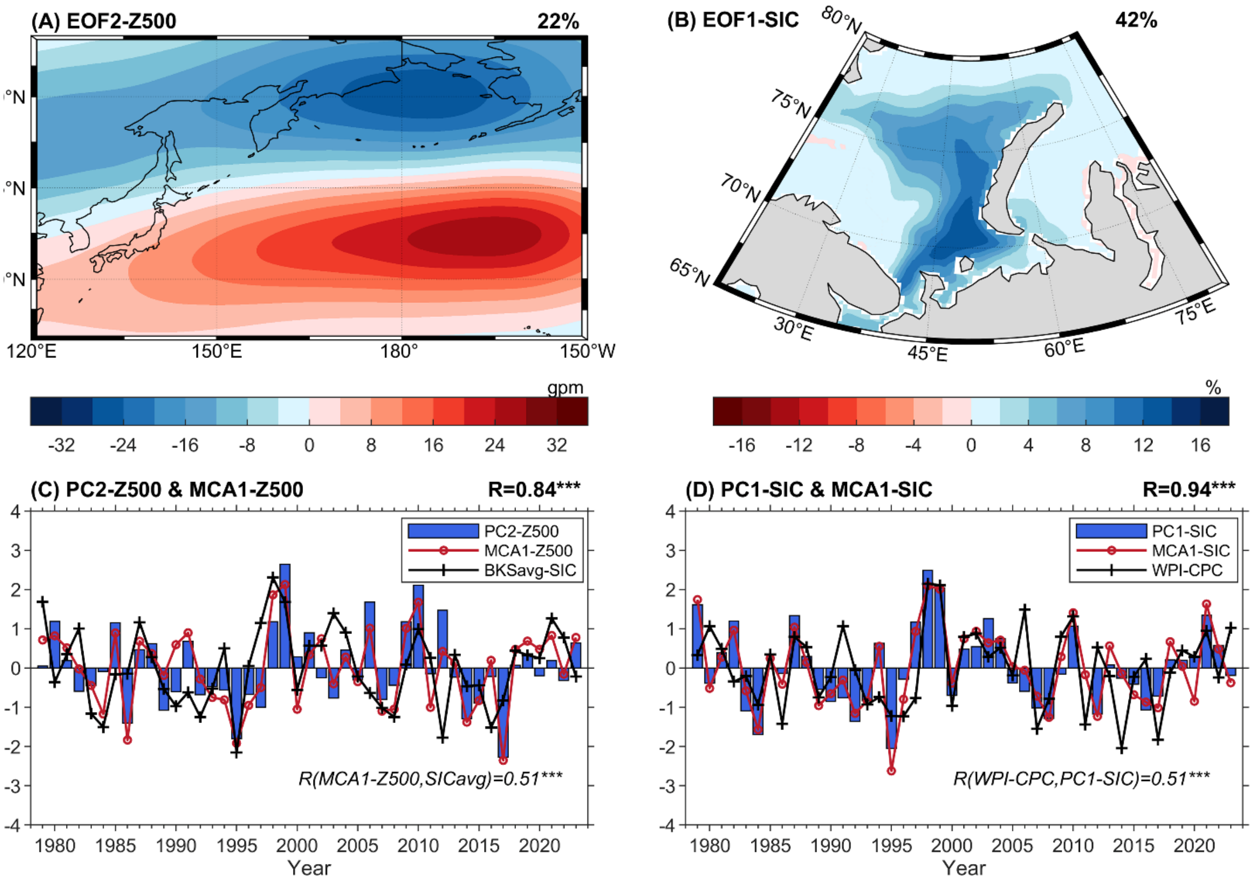

Fig. S3. The results of EOF decomposition for SIC and 500-hPa height (Z500) in FMA during 1979–2023. The second leading EOF mode of Z500 over the western North Pacific region **(A)** and first leading EOF mode of SIC in the BKS **(B)** along with their corresponding principal components and comparisons with the MCA-derived time series **(C and D)**. In (A) and (B), the explained variance corresponding to the EOF mode is displayed in the upper right corner. The correlation coefficients between MCA1-Z500 and the BKS-averaged SIC time series, as well as between the Western Pacific index (WPI) obtained from the Climate Prediction Center (CPC) and the PC1-SIC, are shown in the lower-right corners of panels (C) and (D), respectively. Three asterisks next to the correlation coefficients indicate significance at the 99% confidence level.

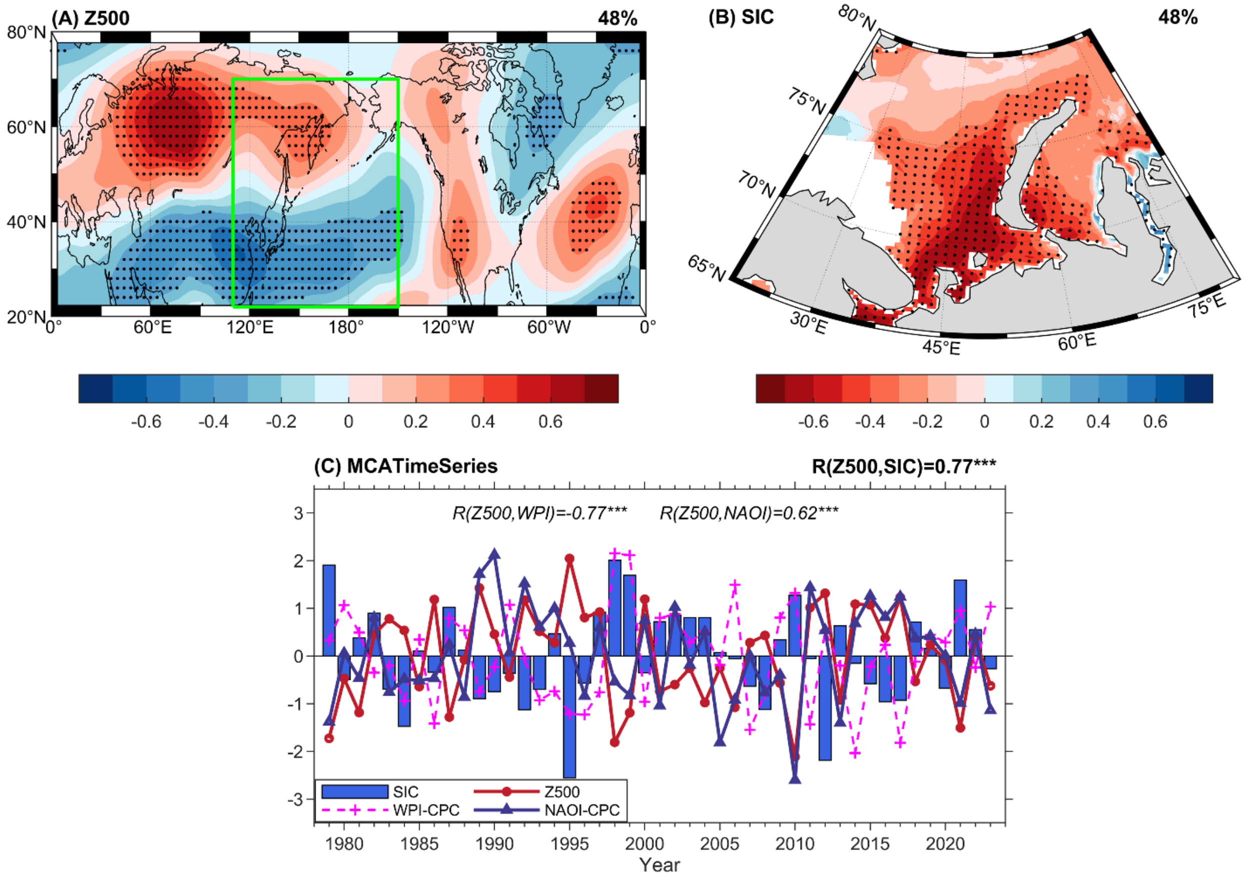

Fig. S4. Maximum Covariance Analysis (MCA) between BKS-SIC and Z500 during FMA over midlatitude regions of the Northern Hemisphere (0°–360°E, 20°–80°N). **(A** and **B)** Heterogeneous correlation maps between local **(A)** Z500 from ERA5 or **(B)** SIC from NSIDC and the temporal coefficient (shown in C) of the first leading MCA (MCA1) mode. **(C)** Normalized expansion coefficients for Z500 (solid red line) and SIC (bar) associated with the MCA1, along with the normalized WPI (magenta dashed line) and North Atlantic Oscillation index (NAOI, blue solid line) from CPC. Stippling in the shading plots indicates statistical significance at the 5% level. The values in the upper-right corners of panels (A) and (B) represent the percentage of variance explained by the MCA1. The green box in panel (A) highlights the WP pattern identified by the MCA1. The upper right corner of panel (C) shows the correlation coefficient between MCA1-Z500 and MCA1-SIC. Correlations between MCA1-Z500 and both WPI-CPC and NAOI-CPC are also shown in panel (C). Three asterisks next to the correlation coefficients indicate significance at the 99% confidence level.

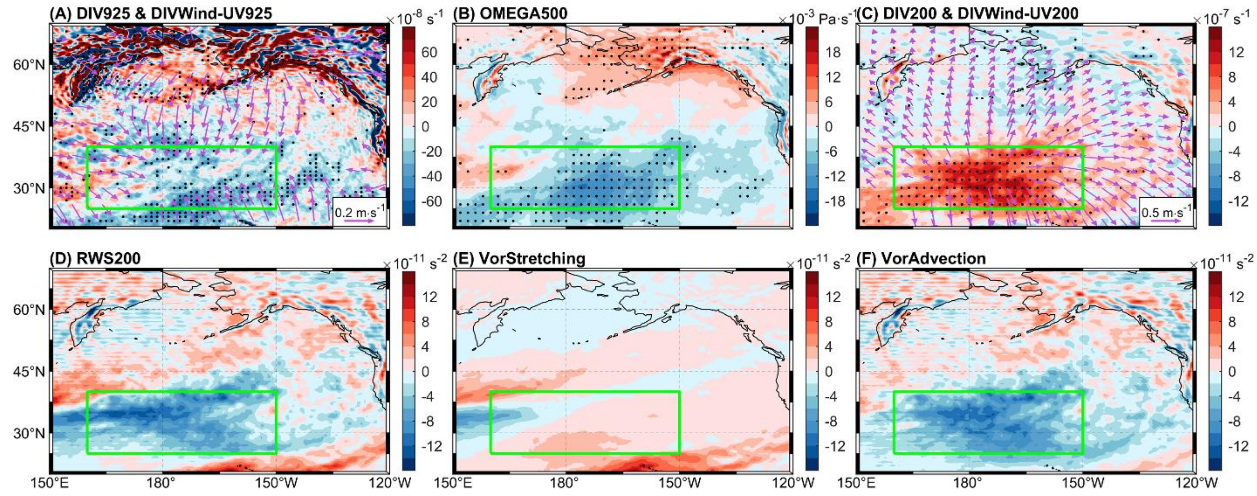

Fig. S5. The WP-associated Rossby wave source and the related physical processes. (A) Regression patterns of divergence (shadings) and divergent winds (vectors) at 925 hPa onto the inverted WPI. **(B)** same as (A), but for vertical velocity at 500 hPa. **(C)** same as (A), but for divergence and divergent winds at 200 hPa. **(D** to **F)** depict the Rossby wave sources associated with the WP at 200 hPa, where (D) represents the total Rossby wave source, while (E) and (F) illustrate the wave sources induced by the vortex stretching term and the vorticity advection term, respectively. Stippling in shading plots indicates statistical significance at the 95% confidence level, only vectors with significance at the 95% confidence level in (A) and (C) are shown. Regions of negative Rossby wave sources caused by divergence are denoted as green boxes.

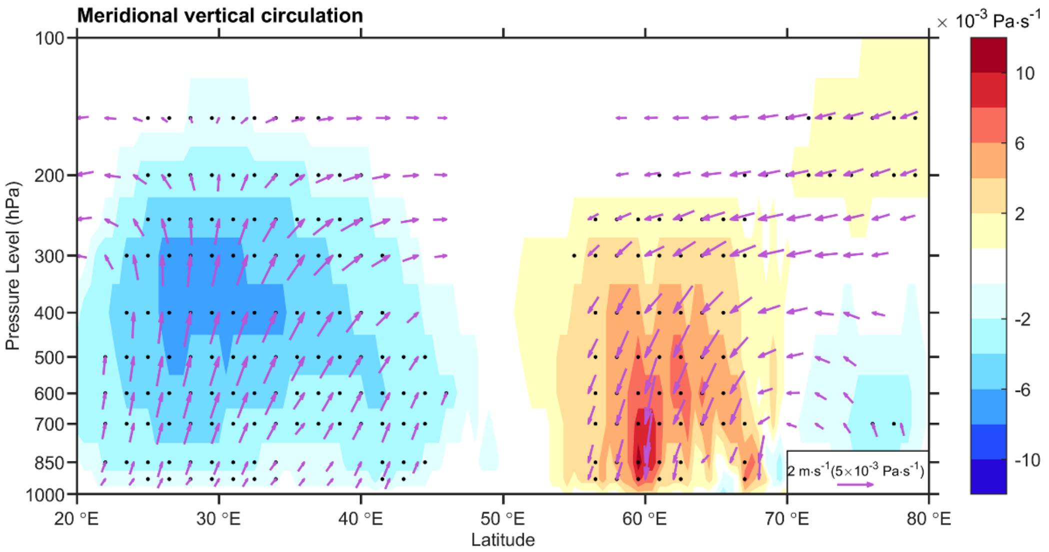

Fig. S6. Regressions of vertical velocity (shadings) and meridional vertical circulation anomalies (vectors) averaged over 160°E to 210°E onto the inverted WPI. Stippling in shading plots indicates statistical significance at the 95% confidence level, only vectors with significance at the 95% confidence level are shown.

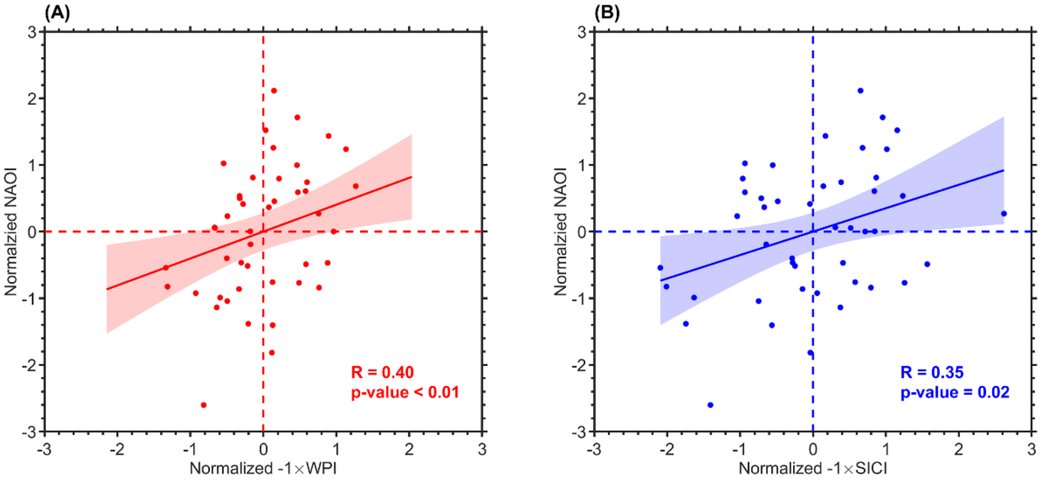

Fig. S7. Scattering plots between the FMA NAOI against WPI (A) and SICI (B), respectively. Their linear regressions (solid lines) as well as the 95% confidence intervals (shadings) are also displayed. The correlation coefficients and their corresponding p-values are annotated in the bottom right corner.

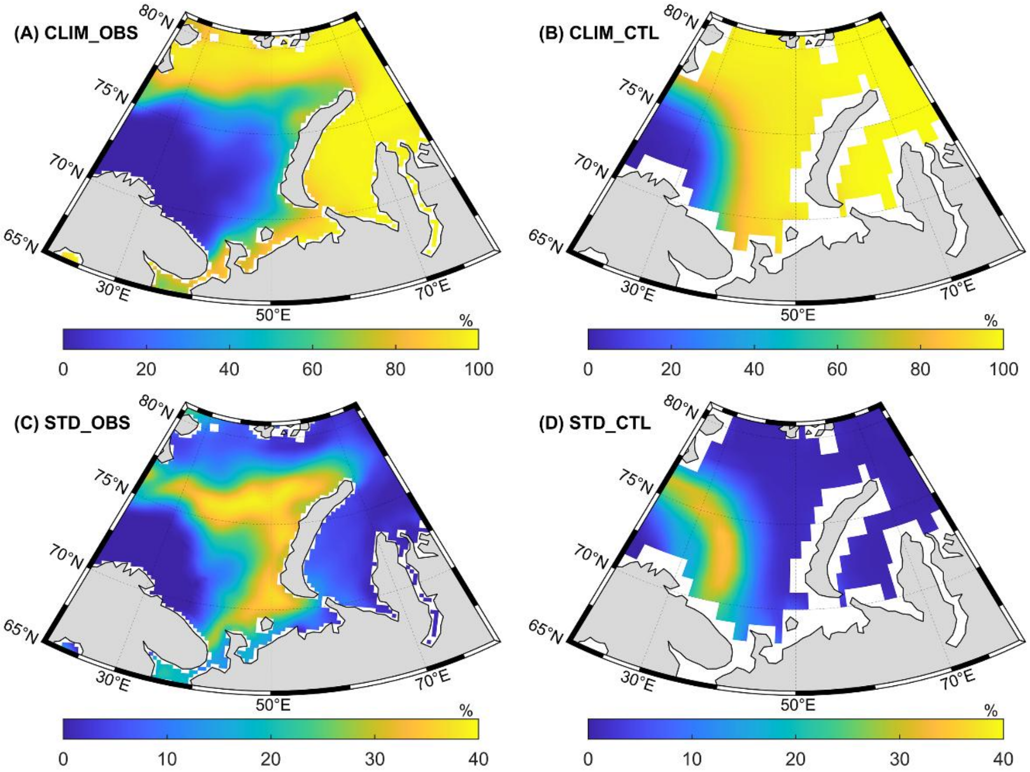

Fig. S8. Comparison of SIC between observations and CESM2 control run. (A) and **(C)** represent the climatological mean and standard deviation of sea ice concentration in FMA during 1979–2023 in the observations. **(B)** and **(D)** are the same as (A) and (C), but for the last thirty years of the CESM2 control run.

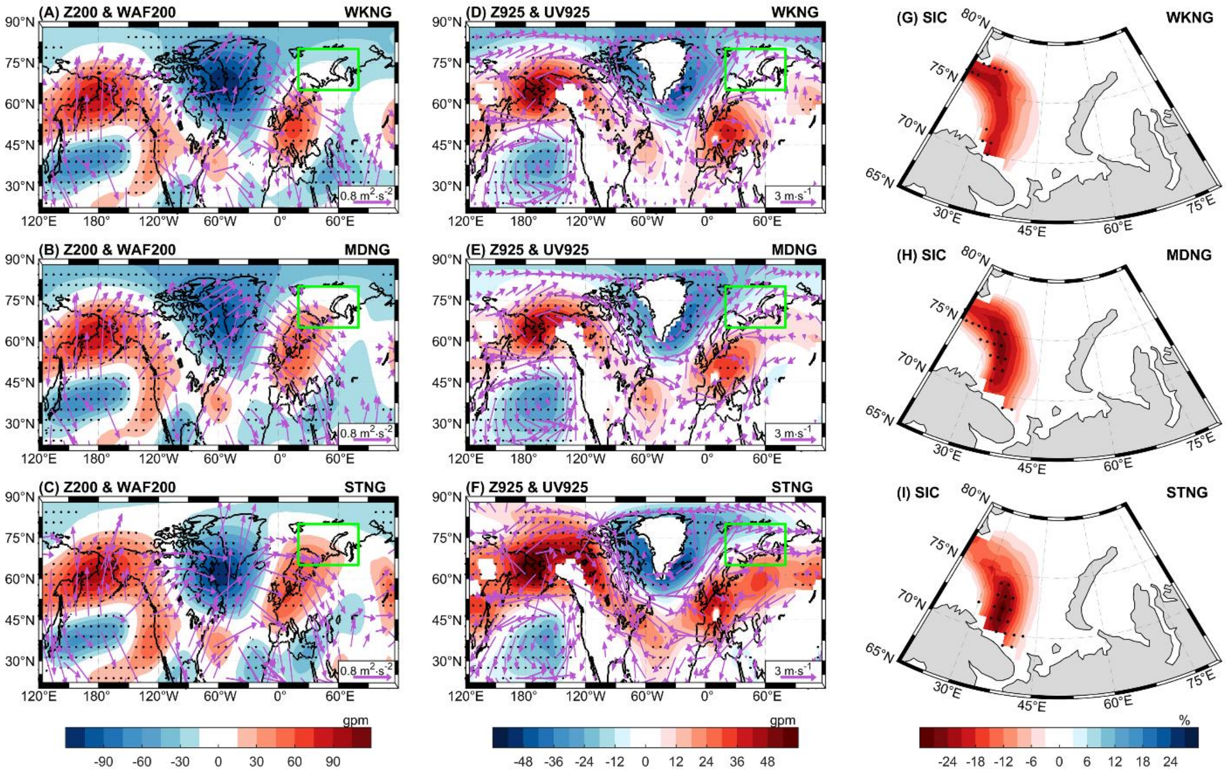

Fig. S9. Atmospheric circulations and SIC anomalies in response to the WP pattern in CESM2 nudging simulations using weak (A, D, and G), moderate (B, E, and H), and strong (C, F, and I) nudging coefficients. Panels (A) to (C) depict the responses of 200-hPa geopotential height (Z200, shading) and wave activity flux (WAF200, vectors), panels (D) to (F) display the responses of 925-hPa geopotential height (Z925, shading) and horizontal wind (UV925, vectors), while panels (G) to (I) illustrate the responses of BKS-SIC. The simulated response to the WP pattern is defined as the ensemble mean difference between the negative and positive WP nudging experiments, consistent with the definition used in Fig. 3. Stippling in the shading plots indicates statistical significance at the 5% level, only significant vectors are shown in (D) to (F). The green boxes denote the BKS region.

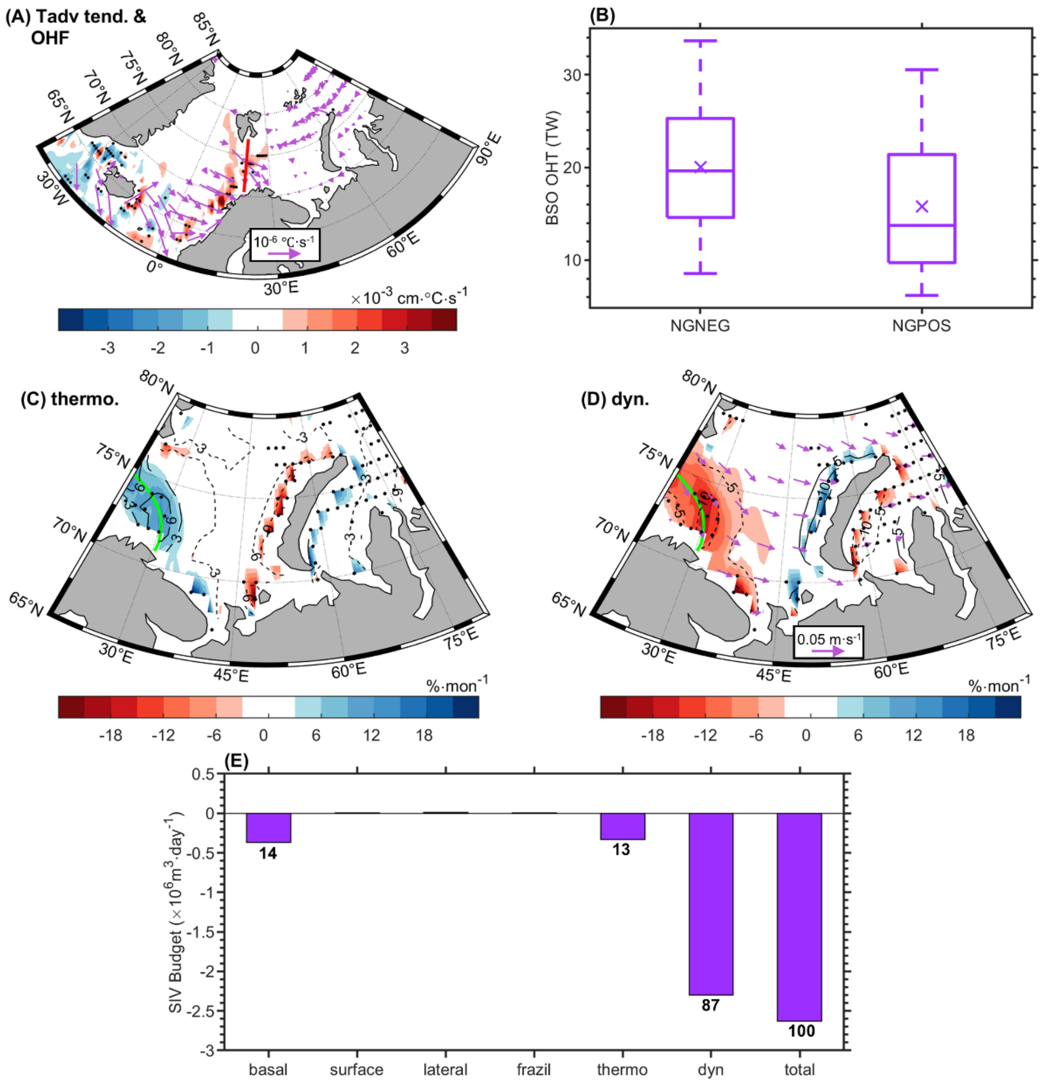

Fig. S10. Ocean heat transport (OHT) and sea ice budget in response to the WP pattern in CESM2 nudging simulations. (A) Responses of vertically integrated temperature advection tendency (Tadv tend., shadings) and horizontal ocean heat flux (OHF, vectors) over the upper 300 meters. **(B)** OHT across the Barents Sea Opening (BSO, read line in A) in the NGNEG and NGPOS experiments. **(C)** and **(D)** are responses of SIC (shadings) and SIT (sea ice thickness, black contours) budget over the BKS due to thermodynamics and dynamics, respectively, with vectors in (D) indicating sea ice velocity. **(E)** Responses of spatially averaged sea ice volume (SIV) budget components in the western Barents Sea (20–45°E, 65–80°N). The numbers at the lower ends of the bars represent the relative contributions (%) of the corresponding components to the SIV tendency anomalies. Stippling of the shading plots indicates statistical significance at the 5% level. In panel (B), the boxes represent the interquartile range (25th–75th percentiles), with the horizontal line and the cross inside each box indicating the median and mean values, respectively. In panels (C) and (D), solid black contours denote positive values, and dashed contours denote negative values, with units of cm/month. The green solid lines in (C) and (D) denote the 15% SIC contours of the ensemble mean across all simulation members.

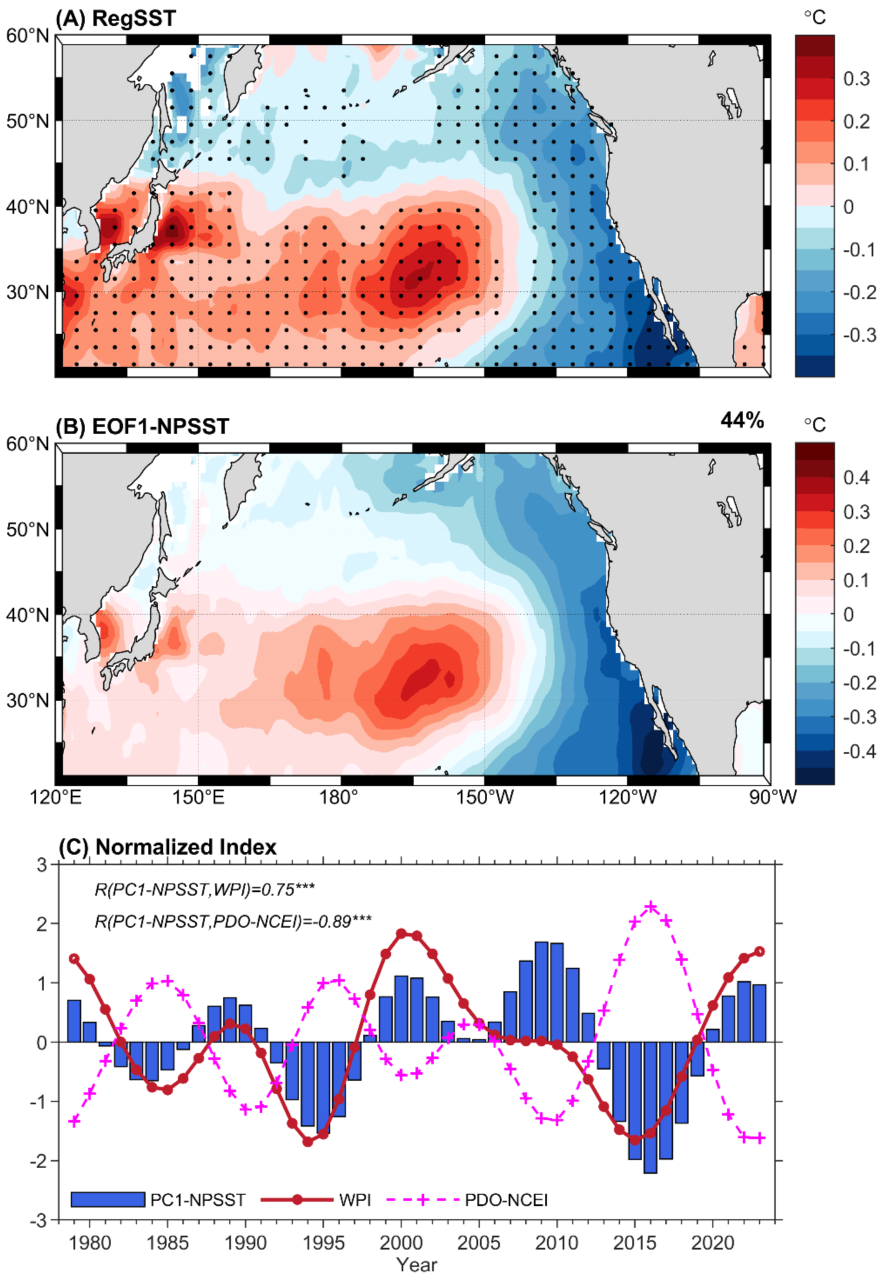

Fig. S11. Decadal-scale connections between WPI and the variability of North Pacific sea surface temperature (NPSST) during FMA. (A) The regression pattern of NPSST anomalies onto WPI on decadal timescales. **(B)** The first leading EOF mode (EOF1) of decadal SST anomalies in the north Pacific region. **(C)** The correlation among the decadal variations of WPI, the principal component of EOF1-NPSST, and the Pacific Decadal Oscillation (PDO) index obtained from the National Centers for Environmental Information (NCEI). Stippling in (A) indicates statistical significance at the 5% level. The number in the upper-right corner of panel (B) indicates the explained variance of EOF1-SST. In panel (C), three asterisks next to the correlation coefficient indicate significance at the 99% confidence level. The decadal variability is extracted using a 9-year low-pass filter.

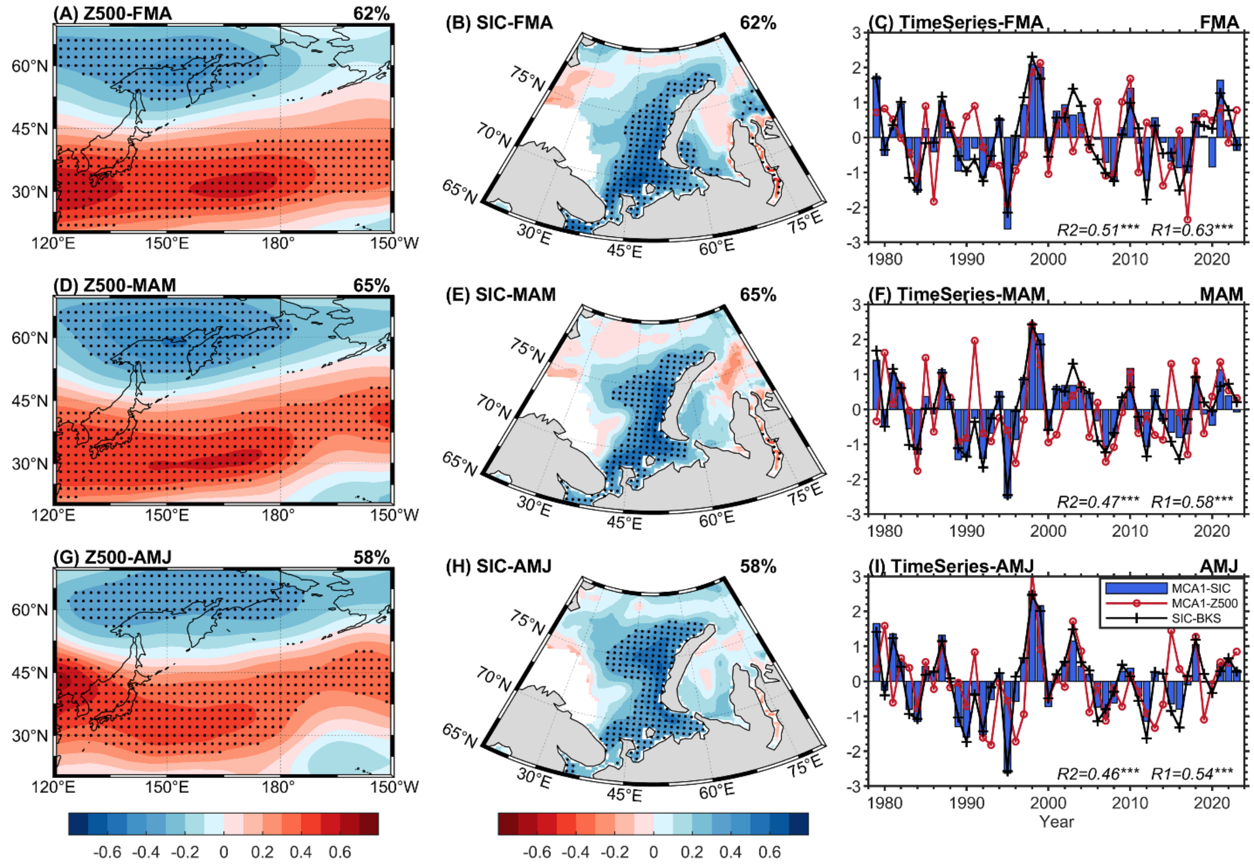

Fig. S12. MCA between SIC in the BKS and Z500 over the western North Pacific region for different spring periods. (A, **D**, and **G)** Heterogeneous correlation maps for Z500 associated with MCA1. **(B**, **E**, and **H)** Heterogeneous correlation maps for SIC associated with MCA1. **(C**, **F**, and **I)** Normalized expansion coefficients for Z500 and SIC associated with MCA1. Panels (A–C), (D–F), and (G–I) show the MCA results for the periods FMA, MAM (March-April-May), and AMJ (April-May-June), respectively. The numbers in the upper-right corners indicate the percentage of variance explained by MCA1. In panels (C), (F), and (I), *R1* represents the correlation between time series of MCA1-Z500 and MCA1-SIC, while *R2* denotes the correlation between time series of MCA1-Z500 and the area-averaged SIC over the BKS. Stippling in the shading plots indicates statistical significance at the 5% level. Three asterisks next to the correlation coefficient indicate significance at the 99% confidence level.

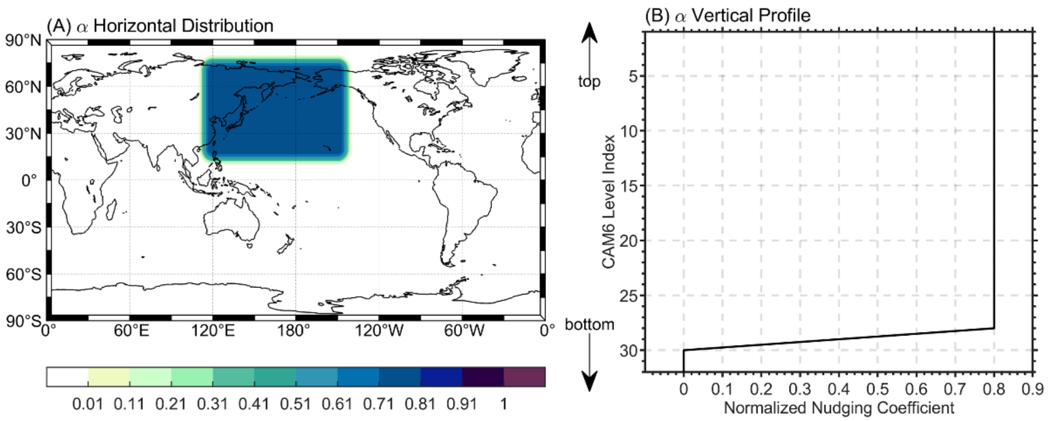

Fig. S13. Nudging coefficients used to constrain the model in the CESM2 experiments of NGPOS and NGNEG. (A) and **(B)** show the horizontal and vertical distributions of the nudging coefficients, respectively.

Table S1. Numerical experiments used in this study.

Exp Name	Nudging or not	Nudging horizontal domain	Nudging vertical levels	Members	Initial Conditions	Length
CLIM-CTRL	No nudging	No nudging	No nudging	1	Branch from CESM2 Historical Run	100 years
NGPOS	Nudging U/V to composites of WP-positive years	120°E–150°W 20°N–70°N	850 hPa to TOA	30	Branch from the last thirty years of CLIM-CTRL	3 months, From 1 Feb to 30 Apr
NGNEG	Nudging U/V to composites of WP-negative years	120°E–150°W 20°N–70°N	850 hPa to TOA	30	Branch from the last thirty years of CLIM-CTRL	3 months, From 1 Feb to 30 Apr
